# Expression of multiple forms of 3'-end variant CCK2 receptor mRNAs in human pancreatic adenocarcinomas

**DOI:** 10.1186/1756-0500-4-131

**Published:** 2011-04-19

**Authors:** Anna Ryberg, Kurt Borch, Hans-Jürg Monstein

**Affiliations:** 1 Division of Clinical and Experimental Medicine, Faculty of Health Sciences, Linköping University, Clinical Microbiology, County Council of Östergötland, S-581 85 Linköping, Sweden; 2 Division of Clinical and Experimental Medicine, Faculty of Health Sciences, Linköping University, Surgery, County Council of Östergötland, S-581 85 Linköping, Sweden

**Keywords:** CCK2-receptor mRNA expressions, CCK2i4sv splice variant mRNA, CCK2-receptor and gastrin mRNA co-expression, amplified mRNA, human pancreatic adenocarcinoma

## Abstract

**Background:**

Two main types of receptors for gastrin and cholecystokinin (CCK) have been cloned and identified. CCK1 (CCK-A) receptors are expressed in the pancreas, the gallbladder, and parts of the brain, while CCK2 (CCK-B/gastrin) receptors (CCK2R) are expressed in gastric glands and in most of the brain. A splice variant of the CCK2R designated CCKRi4sv (CCK-C), which is constitutively expressed in human pancreatic cancer cells, has also been described. The purpose of the present investigation was to study CCK2R, CCK2i4svR, and gastrin mRNA expression in human pancreatic adenocarcinoma on the assumption that co-expression of CCK2R and gastrin or constitutive CCK2i4svR mRNA expression plays a pivotal role in the progression of pancreatic cancer.

**Findings:**

PCR amplification using CCK2R specific primer-pairs, followed by ethidium-bromide stained agarose gel electrophoresis revealed the expression of wild-type CCK2R mRNA in 12 of 17 biopsy specimens. A CCK2R intron 4 specific nested PCR assay revealed that CCK2i4svR mRNA was expressed in only one of the biopsy specimen. The authenticity of PCR amplicons was confirmed by cloning of selected amplicons and DNA sequence analysis. Moreover, we found that hitherto undescribed multiple forms of 3'-end variant CCK2R mRNAs with various deletions in the retained intron 4 and exon 5, tentatively generating truncated proteins, were expressed in the pancreatic adenocarcinomas.

**Conclusion:**

Cloning and DNA sequencing of selected amplicons revealed that CCK2R and multiple CCK2i4svR-like mRNAs are expressed in human pancreatic adenocarcinoma. The originally described CCK2i4svR mRNA was only expressed in one of 17 tumours and appears to be rarely expressed in pancreatic adenocarcinoma. We report that CCK2R- and gastrin mRNA co-expression may play a role in a portion, but not in all of these tumours, and that aberrant splicing takes places in these tissues generating multiple forms of 3'-end variant CCK2R mRNAs.

## Background

Two main types of receptors for gastrin and cholecystokinin (CCK) have been cloned and identified [1-4], the CCK1-receptor (formerly named CCK-A receptor) which is expressed in the pancreas, the gallbladder and parts of the brain, whereas the CCK2-receptor (formerly named CCK-B/gastrin receptors) is expressed in gastric glands and in most of the brain [1-5]. Both types of receptors are known to signal by coupling to G proteins and to a have growth-stimulating effect [6,7]. CCK receptor expression has been studied extensively in various tissues and cell-lines including normal and diseased human pancreatic tissue. However, it has been suggested that CCK1- and CCK2-receptors are differently expressed in normal human pancreas and pancreatic cancer cells [5,8-14]. The significance of this remains still unknown.

The relationship between CCK2-receptor (CCK2R) mRNA, gastrin mRNA, and gastrin peptide expression has been studied by several authors in the past [11,15-17]. In normal human pancreas low levels of gastrin mRNA were detected, but gastrin peptides were not present. In contrast, pancreatic cancer cells and cell-lines expressed high levels of CCK2 and gastrin mRNA as well as gastrin peptides [15,17,18]. Gastrin has been shown to stimulate the growth of human pancreatic adenocarcinoma and pancreatic cancer cell-lines. Based on these findings, it was suggested that human pancreatic adenocarcinomas might be stimulated by an autocrine loop involving co-expression of CCK2R and gastrin [7,17-19]. Apparently, an antisense oligonucleotide directed against gastrin mRNA inhibited growth of human pancreatic cancer cells up to 88% inhibition in a dose dependent fashion, supporting the role of gastrin as a growth regulatory peptide for human pancreatic cancer [20]. In contrast, Ohlsson and co-workers reported that gastrin had no growth promoting effect on human pancreatic cancer cell-lines derived from pancreatic cancer biopsy specimens obtained at surgery [21].

A CCK2R mRNA splice variant expressed in human pancreatic cancer was originally reported by Smith and co-workers [20,22]. These authors demonstrated that the novel splice variant mRNA is expressed exclusively in human pancreatic cancer and is not present in normal human pancreas. Based on their findings, the term CCK-C or "cancer" receptor has been proposed [22]. Hellmich and co-workers [23] reported on the identification and isolation of a novel splice variant of the human CCK2R expressed in human colorectal cancers, but not in normal colonic mucosa adjacent to the cancer. Molecular cloning and DNA sequencing revealed that the novel splice variant mRNA was identical to the CCK-C receptor and was designated CCK2i4svR (for intron 4-containing splice variant receptor), and codes for a CCK2R protein that contains 69 additional amino acids. This protein was identical to the CCK-C receptor [20,22]. Subsequent studies revealed that the novel CCK2i4svR is constitutively expressed in an agonist-independent manner by a Src tyrosine kinase-dependent pathway [23-25]. Ding and co-workers [26] confirmed the expression of the CCK2i4svR mRNA in pancreatic cancer and no expression in normal pancreatic tissue. Moreover, these authors reported that CCK2i4svR mRNA expression is due to low levels of a U2 splicing factor creating aberrant splicing in malignant cells [26].

The purpose of the present investigation was to study the expression/co-expression of wild type CCK2R, CCK2i4svR, and gastrin mRNA in human pancreatic adenocarcinoma by means of RT-PCR and DNA sequence analysis of selectively cloned PCR amplicons. We report that CCK2R- and gastrin mRNA co-expression or constitutive CCK2i4svR mRNA expression occurs only in a part of these tissues.

## Methods

### Tissue samples

Anonymous archival pancreatic tumour tissue samples from 17 deceased patients with histopathological diagnosed pancreatic ductal adenocarcinoma were analysed. The study was approved by the Regional Ethics committee in Linköping, Sweden (Dnr. M38-06). A tissue block (minimum 5 × 5 × 5 mm) had been excised from the primary tumour immediately after surgical resection (14 cases) or from extra-pancreatic tumour growths when radical resection was not possible (peritoneal growth in two cases and liver metastasis in one case). Care was taken not to contaminate tissue samples with bile or contents from the stomach or intestine. The samples were stored at -80°C in RNA storage solution (Ambion, Austin TX, USA) until RNA isolation.

### Isolation of total RNA and poly(A)+ RNA

Total RNA was isolated from the tissue using the RNeasy Midi kit (Qiagen, Hilden, Germany). A cube of approximately 4 mm square was cut out from the tissue sample and placed in 1.5 ml RTL buffer, containing β-Mercaptoethanol. Three 3.2 mm Chrome-Steel beads (BioSpec Products, Inc. Bartlesville OK, USA), prewashed in RTL buffer, were added to each tube. The tissue samples were homogenized using the Mixer Mill 300 (Qiagen, Hilden, Germany) for 5 min at 30 Hz, the tube racks were rotated 180° and then homogenized for additional 5 min at 30 Hz. Normally, this was sufficient for complete homogenisation of the tissue, but additional shaking was needed for some samples. After the homogenisation, total RNA was isolated according to the manufacturer's instructions and eluted in two times 150 μl RNase-free water. Total RNA samples were concentrated using the RNeasy MinElute Cleanup kit (Qiagen, Hilden, Germany) and eluted in 20 μl RNase-free water. Subsequently, total RNA was treated with a TURBO DNase kit following the protocol (Ambion, Austin TX, US). Poly(A)+ RNA (mRNA) was isolated from total RNA using an automated BioRobot M48 workstation and the MagAttract Direct mRNA M48 kit (Qiagen, Hilden, Germany) and concentrated as described above. mRNA concentrations were measured using a BioPhotometer v. 1.32 (Eppendorf, Hamburg, Germany).

### RNA amplification

100 ng of the poly(A)+ RNA was amplified twice in independent reactions using the MessageAmp II aRNA kit (Ambion, Austin TX, USA). The amplification is based on the Eberwine protocol [27] where a T7-promotor is incorporated into the ds-cDNA during reverse transcription from the isolated mRNA. RNA is then amplified using T7 RNA polymerase. The amplification is linear and a possible bias introduced in amplified RNA is very low [28]. Before storage at -80°C, the amplified antisense-RNA (aRNA) was quantified in triplicate measurements, using a ND-1000 spectrophotometer (Nanodrop Technologies, Wilmington DE, USA).

### aRNA purity and ss-cDNA synthesis

The purity of RNA (absence of contaminating DNA) was analysed by a commercially available β-actin control PCR (Human β-actin Control Amplimer Set from BD-Biosciences, Palo Alto CA, USA; Table 1) using PCR condition No. 1 as specified (Table 2). The abundance of β-actin pseudogenes in the human genome makes it a suitable gene for control of DNA contamination [29]. The amplified aRNA contaminated with DNA was treated with the TURBO DNase kit (Ambion, Austin TX, USA), following the rigorous protocol, and again validated by the β-actin control PCR.

**Table 1:** Primers used in PCR amplification.

Name	Sequence, 5' to 3' direction	Size (bp)	Start position	Reference
(1) hCCK-BR/I..SE	5'-GTGGCCTACGGGCTTATCTCTCGCGAGCTCTACTTA	358/565	709	[GenBank:AF239668]
(2) hCCK-BR/II.AS	5'-ACGTGTTGGCACTATAAACTGGCAACCAACACAG	1240		
(3) CCKBRseq.SE1	5'-AGGGTCCGAAACCAAGGC	441/234	787	
(4) CCK-BR.AS2	5'-AACGATCACCAGCAACATTCGC		1206	
(5) CCK-BRwt.SE	5'-CGGACTACTCATGGTGCCCTAC	832/523/317	549	
(6) CCK-BRwt.AS	5'-GCCAACCGCGCCAGTCTCAG		1052	
(7) CCK-BRi4sv.AS	5'-CCATTTCCAGCTTCCTTCTCA	691/383	911	

Gas1.SE	5'-CAGCAGCCAGATGCACCCTTAGGTACAG	386/256	6370	[GenBank:M15958.1]
Gas1.AS	5'-GGCTAGGCTCTGAAGCTTGGTTCTAGGATTGTTAG		6755	

β-Actin.SE	Human β-Actin Control Amplimer set	838	BD Biosciences, Palo Alto, CA. USA	
β-Actin.AS				

**Table 2:** PCR conditions.

PCR No.	Initial denaturation	Denaturation	Annealing	Extension	Cycles	Final extension
1	95°C, 15 min	95°C, 30 s	65°C, 30 s	72°C, 1 min	35	72°C, 10 min
2	95°C, 15 min	95°C, 30 s	65°C, 30 s	72°C, 1 min	30	72°C, 10 min
3	95°C, 15 min	95°C, 30 s	55°C, 30 s	72°C, 1 min	30	72°C, 10 min
4	95°C, 15 min	95°C, 30 s	52°C, 30 s	72°C, 1 min	30	72°C, 10 min

Single stranded complementary DNA (ss-cDNA) was synthesized using SuperScript III ss-cDNA synthesis system (Invitrogen, Carlsbad CA, USA), hexamer primers and approximately 100 ng poly(A)+ RNA template. The ss-cDNA synthesis was repeated twice for each aRNA sample, resulting in six cDNA pools from each biopsy. The cDNA synthesis reaction was also validated using the β-actin PCR.

### PCR amplification

**Figure 1:**
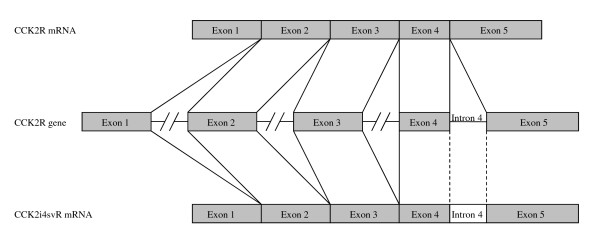
**Schematic drawing (not to scale) of the CCK2R gene exon/intron arrangement **[3], **the CCK2R mRNA (wt) **[3], **and CCK2i4svR splice variant mRNA **[23].

**Figure 2:**
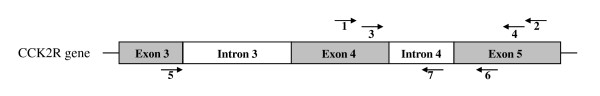
**Schematic drawing (not to scale) of the CCK2R gene 3'-region corresponding DNA sequences of exon 3 to exon 5**. Arrows indicate the position of the CCK2R primers No. 1-7 described in Table 1.

The expression of CCK2R, CCK2i4svR, and gastrin mRNAs was assessed by PCR amplification of ss-cDNA. Amplifications were carried out in a final reaction volume of 25 μl using thin-walled reaction tubes, 1x HotStarTaq Master Mix kit (Qiagen, Hilden, Germany), and a PTC-100 Programmable Thermo Controller (MJ Research; SDS Biosciences, Falkenberg, Sweden). Primer sequences, expected amplicon sizes, and PCR amplification conditions used are given in table 1 and table 2. A schematic drawing of the CCK2R gene, CCK2R, and CCK2i4svR mRNAs is shown, including the position of the primers used in the present study (Figure 1 and 2).

To evaluate the expression of the gastrin gene, PCR using the Gas1.SE (located in exon 2) and Gas1.AS (located in exon 3) primer-pair (Table 1), and condition No. 1 (Table 2), was carried out. The expected sizes of ss-cDNA or genomic DNA derived gastrin amplicon are 256 bp and 386 bp (including intron 2), respectively.

A nested PCR assay was used for validation of expressed CCK2R mRNA variants. In the first amplification step, primer 1 and 2 (hCCK-BR/I.SE and hCCK-BR II.AS) located in exon 4 and 5 (Figure 2), and PCR amplification condition No. 2 were used. Amplicons were purified using the GFX PCR DNA gel band purification kit (GE-Health Care, Uppsala, Sweden), followed by a second PCR using 1 μl purified amplicon, primer 3 and 4 (CCKBRseq.se1 and CCKBR.as2), and PCR amplification condition No. 3.

To confirm the expression of CCK2i4svR mRNA, a semi-nested PCR assays were carried out. Firstly, PCR amplification was carried out using primer 5 (CCKBRwt.SE) located in exon 3 and primer 6 (CCKBRwt.AS) located in exon 5 (Figure 2) and PCR condition No. 4. PCR amplicons were purified as described above. Genomic DNA or ss-cDNA retaining intron 3 and 4 yields an amplicon of 832 bp, ss-cDNA corresponding to CCK2i4svR (retaining exon 4) an amplicon of 523 bp, and ss-cDNA corresponding to the CCK2R an amplicon of 317 bp. Semi-nested PCR was carried out using 1 μl purified amplicon and primer 5 (CCKBRwt.SE) and primer 7 (CCKBi4svR.AS) located in intron 4 (Figure 2), and PCR condition No. 2. Genomic DNA or ss-cDNA retaining intron 3 yields an amplicon of 691 bp and ss-cDNA corresponding to CCK2i4svR an amplicon of 383 bp (Table 1). Amplicons were visualised on an ethidium-bromide stained 1.5% agarose gel. Genomic DNA was included in all PCR amplification assays to visualise differences in amplicon sizes compared to ss-cDNA derived amplicons. No template controls were included in all PCR assays.

### Cloning of CCK2-receptor amplicons and DNA sequence analysis

Amplicons were cloned using the TOPO-TA cloning kit (Invitrogen, Carlsbad CA, USA). Positive clones were selected and plasmid DNA was prepared by means of multiple displacement amplification [30] using an Illustra GenomiPhi V2 DNA amplification kit as recommended by the manufacturer (GE-Healthcare, Uppsala, Sweden). DNA sequence analysis was carried out without further purification [31] by a customer DNA sequencing service (Eurofins MWG Operon GmbH, Martinsried, Germany). Generated DNA sequences were aligned, edited, and compared with CCK2R [GenBank:NM_176875.2] and CCK2i4svR [GenBank:AF441129] which were retrieved from the NCBI Entrez Nucleotide database [http://www.ncbi.nlm.nih.gov/nucleotide]. The sequences were compared by aligning using ClustalW [EMBL-EBI ClustalW2 http://www.ebi.ac.uk/tools/clustalw2].

## Results

### RNA and ss-cDNA synthesis

**Figure 3:**
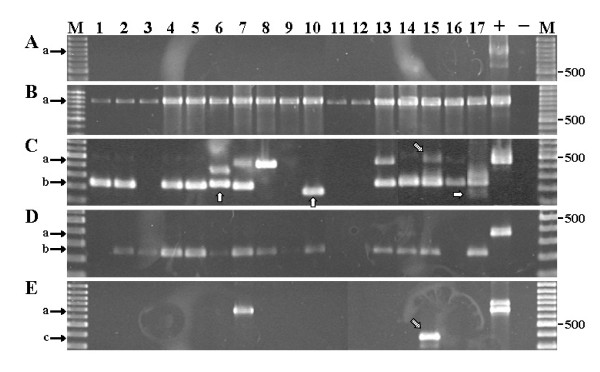
**Representative Ethidium-bromide stained agarose gel visualising PCR amplicons derived from 17 archival biopsy specimens (lane 1 to 17)**. A) Quality control of total RNA (free of contaminating genomic DNA), and B) single-stranded cDNA by means of β-actin PCR amplification after DNase treatment. C) Visualised amplicons from nested CCK2R, D) gastrin, and E) CCK2i4svR specific PCR assays as described in methods. The position of the 500 bp size marker in each 100 bp ladder (lane M) is indicated. Black arrows in the left margin indicate the position of a) amplicons derived from genomic DNA or cDNA retaining introns, b) wild-type CCK2 receptor and gastrin mRNA amplicons, and c) CCK2i4svR splice variant amplicons derived from ss-cDNA. White arrows (lane 6, 10, and 17) indicate confirmed CCK2 receptor splice variants by DNA sequencing. Stripped arrows indicate the position of the CCK2i4svR amplicon in sample No. 15. In all amplification assays human genomic DNA (+) was included as control. (-) NTC (no DNA template added to the master mix).

The amount of amplified mRNA obtained from each biopsy ranged from ~2 to 80 μg with purity of A_260/280 _1.9 for most of the samples (ranging from 1.3-2.06). In average, mRNA amplification yielded 6 μg aRNA from 60 ng mRNA input. The absence of human genomic DNA after DNase treatment was confirmed by means of β-actin amplification prior to the aRNA amplification step (Figure 3A). Subsequently, aRNA (a maximum volume of 8 μl, but not more than 500 ng) was used in ss-cDNA synthesis, which was confirmed by β-actin PCR amplification (Figure 3B).

### Expression of CCK2-receptor and gastrin mRNAs

To determine the expression of CCK2R and gastrin mRNAs, ss-cDNA derived from isolated human pancreatic adenocarcinoma biopsy specimens were analysed by PCR using CCK2R and gastrin specific primers. Ethidium-bromide stained agarose gel electrophoresis revealed the presence of an approximately 250 bp amplicon corresponding to a wild-type (wt) CCK2R mRNA in 12 of 17 (71%) biopsy specimens (Figure 3C, lane 1, 2, 4-7, 10, 13-17). Furthermore, in 10 of these biopsies (59%), both weak and strongly visible amplicons of approximately 400-500 bp were present, indicating that the CCK2R and CCK2i4svR-like mRNAs were co-expressed (Figure 3C, lane 1, 2, 4-7, 13-16). In one biopsy each, a single amplicon corresponding to CCK2i4svR-like or CCK2R mRNA, respectively, was present (Figure 3C, lane 8, 10). In four biopsy specimens, no amplicons corresponding to CCK2R or CCK2i4svR-like mRNAs were detected (Figure 3C, lane 3, 9, 11, 12). In 13 of 17 (76%) biopsy specimens, PCR amplicons corresponding to gastrin mRNAs were present (Figure 3D, lane 2-10, 13-15, 17). CCK2R (wt) and gastrin mRNAs were co-expressed in 10 of 17 (59%) biopsy specimens (Figure 3C and 3D, lane 2, 4-7, 10, 13-15, 17), and in one case CCK2i4svR-like and gastrin mRNA were co-expressed (Figure 3C and 3D, lane 8). Similarly, CCK2R, -CCK2i4svR-like and gastrin mRNAs were co-expressed in eight biopsy specimens (Figure 3C and 3D, lane 2, 4-7, 13-15).

To confirm the expression of a CCK2i4svR-like mRNA, a semi-nested PCR assay was carried out using primers targeting exon 3 and 5, and intron 4 (Figure 2, table 2). Surprisingly, only one of seven tentative CCK2i4svR-like amplicons appears to correspond to the originally described CCK2i4svR-mRNA (Figure 3E, lane 15). One amplicon revealed an unexpected size of approximately 800 bp corresponding to a CCK2R mRNA retaining intron 3 and 4 (Figure 3C, lane 7).

### Cloning and DNA sequence analysis

**Figure 4:**
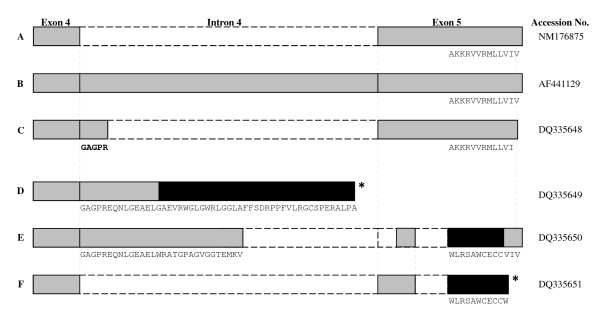
**Schematic drawing (not to scale) of cloned and sequenced CCK2R variants**. A) grey boxes correspond to amino acid sequences (exon 4 and 5) of the wt CCK2R [GenBank:NM176875] and B) CCK2i4svR splice variant receptor (exon 4, intron 4, exon 5) [GenBank:AF441129]. C) CCK2R splice variant consisting of exon 4, the first five N-terminal amino acids of intron 4, and one base truncation at the C-terminal end in exon 5 [GenBank:DQ335648]. D) CCK2R splice variant consisting of exon 4, a deviating and truncated intron 4 (black boxes indicate that a frame-shift mutation has occurred generating putative amino acids deviating from the wild-type sequences). [GenBank:DQ335649]. E) [GenBank:DQ335650] and F) [GenBank:DQ335651] CCK2R splice variants with deviating amino acid sequences in intron 4 and exon 5 compared to CCK2R (wt) and CCK2i4svR. Amino acid sequences deviating from previously reported CCK2R and CCK2i4svR sequences are indicated using the single letter amino acid code. The positions of in frame stop codons yielding tentatively truncated CCK2 receptors in D) and F) are indicated by an asterix.

DNA sequence analysis was carried out on selected and cloned CCK2R amplicons. Sequences were aligned in ClustalW and compared with CCK2R sequences [GenBank:NM_176875.2] and CCK2i4svR [GenBank:AF441129] (Figure 4). DNA sequence analysis revealed that the two amplicons derived from sample No. 6 of 247 bp and 358 bp, respectively, (Figure 3C), correspond to the CCK2R long form (Figure 4C) [22] and a new CCK2i4R splice variant carrying a deletion of 83 bp in the retained intron 4. *In silico *translation of the nucleotide sequence generates a tentatively truncated CCK2R-protein (Figure 4D). Semi-nested PCR revealed the absence of a CCK2i4svR-specific amplicon (Figure 3E), indicating that the cloned CCK2R sequences might be attributed either to a new CCK2R variant lacking target sequence for primer 7 (CCK-BRi4sv.AS, table 1), or to contaminating genomic DNA.

The 180 bp amplicon derived from sample No. 10 represents another, hitherto undescribed CCK2R splice-variant having a 162 bp deletion in the 3'-part of intron 4 and two deletions in the 5'-part of exon 5. *In silico*-generated amino acid sequence deviate from the originally described CCK2i4svR sequence (Figure 4E). Sequence analysis of the 137 bp amplicon corresponding to the weak band visualised in sample No. 17 (Figure 3C) revealed a 97 bp deletion in exon 4, encoding a tentatively truncated CCK2R variant protein (Figure 4F). The DNA sequences have been reported to the NCBI Entrez Nucleotide database [GenBank:DQ335648], [GeneBank:DQ335649], [GenBank:DQ335650] and [GenBank:DQ335651].

## Discussion

Gastrin is known to stimulate gastric acid secretion and growth of the acid-producing mucosa [32,33]. It has also been shown that gastrin stimulates pancreatic growth [34,35]. If the current view of gastrin as a trophic regulator of the pancreas is correct, the receptor for gastrin (CCK2R) should be expected to be expressed in pancreas. By means of Northern blot and RT-PCR analysis, we and others have shown that the CCK2R mRNA is expressed in adult human pancreas [5,36,37]. Furthermore, it has been shown that CCK2R mRNA, gastrin mRNA and its corresponding proteins are expressed in human pancreatic adenocarcinoma [15,17]. The current view is that co-expression of gastrin and CCK2R (wt) regulates growth of human pancreatic cancer in an autocrine fashion [17,18,20]. As judged from RT-PCR analysis in the present study, CCK2R (wt) and gastrin mRNA were both expressed in 10 of 17 biopsy specimens. It was claimed in a previous study that nearly all human pancreatic carcinomas studied expressed both CCK2R (wt) and gastrin mRNA [17]. It could be argued that the RT-PCR analysis used herein is not sensitive enough to detect low levels of CCK2R- and gastrin mRNA expression. To overcome this potential problem we used an initial mRNA amplification step which is commonly used in chip-array based gene expression studies [27]. Thus, the present data suggest that co-expression may play a role in a portion but not in all of these tumours.

Smith and co-workers showed that antisense oligonucleotide to gastrin mRNA did not completely inhibit growth of human pancreatic cancer [20]. This would indicate that beside CCK2R/gastrin autocrine or paracrine growth loops, hitherto unknown CCK2R or gastrin induced pathways might be involved in stimulation of pancreatic cancer growth, which could explain the absence of co-expressed CCK2R (wt) and gastrin mRNAs in our study. Constitutive expression of an alternatively spliced CCK2i4svR mRNA activating intracellular signal pathways has been described [22,23,38] indicating that CCK2i4svR mRNA expression is a contributing factor in human pancreatic cancer. However, by means of RT-PCR analysis we found rather unexpectedly that only one of 17 pancreatic adenocarcinoma biopsy specimens generates amplicons corresponding to CCK2i4svR mRNA (Figure 3, lane 15).

On the other hand, molecular cloning and DNA sequence analysis of selected CCK2i4svR amplicons as described in the present study revealed a complex CCK2R mRNA expression pattern in the human pancreatic adenocarcinomas. Beside CCK2R, CCK2R long form, and CCK2i4svR mRNA, we present evidence for the expression of three novel, hitherto undescribed CCK2R variant mRNAs. It is tempting to speculate that CCK2i4svR mRNA and these novel CCK2R mRNA variants are generated by an aberrant splicing mechanism as described by Ding and co-workers [26]. Using an *in vitro *mini-gene expression, transcription, and splicing system and the MIA PaCa-2 pancreatic cancer cell-line, they were able to gain insight into the molecular mechanism of missplicing of CCK2R pre-mRNA in these cells. Re-engineering of the non-optimal 3'-splice site in intron 4 of the CCK2R pre-mRNA, but not the 5'-splice site of this intron to the consensus sequence markedly improved the splicing of the primary transcript in the MIA PaCa-2 pancreatic cancer cell-line. Moreover, evidence was provided that the expression of the U2AF35 splicing factor which specifically recognizes the 3-end splice site, essential for correct pre-mRNA splicing, is significantly lower in human pancreatic adenocarcinoma and pancreatic cancer cell-lines than in normal pancreatic tissue [26].

In this context, it is interesting to note that the three novel CCK2R splice variants described in the present study appear to be generated by missplicing at the 3'-splice site in intron 4 (Figure 4D-F). In addition, two of these novel CCK2R variants revealed alternative splicing in exon 5, tentatively generating CCK2svR proteins with an alternative carboxyl terminus (Figure 4E, F). CCK2R internalisation and intracellular trafficking have been shown to be influenced by a number of different structural determinants of the receptor, including the carboxyl terminus [39,40]. Thus, it is conceivable that an altered carboxyl terminus generated by alternative splicing of exon 5 may have an impact on internalisation and intracellular trafficking of CCK2svR receptors. In lack of experimental evidence, we can only speculate that these novel 3'-end variant CCK2R mRNAs could be translated into CCK2svR-like proteins with similar functions in human pancreatic cancer growth as described for the original CCK2i4sv receptor [22,23,38].

Aberrant alternative splicing of pre-mRNA, also referred to as illegitimate or ectopic splicing [41], has been found to be associated with various diseases, including cancer. The discovery of cancer-specific, alternatively spliced isoforms has prompted interest in their potential use as disease biomarkers, both at the mRNA and protein level. Thus, it has been suggested that aberrant alternative splicing may provide a potential source for new diagnostic and prognostic tools [42,43]. Deciphering the mechanisms underlying aberrant alternative splicing of CCK2R pre-mRNA and a better understanding of the biological function (if any) of its cognate proteins may possibly lead to elucidation of human pancreas transformation mechanisms and cancer development.

## Conclusion

By means of PCR amplification and cloning of selected amplicons we show that aberrant splicing takes place in pancreatic adenocarcinomas generating multiple forms of 3'-end variant CCK2R mRNAs. Moreover, we find that the originally described CCK2i4svR mRNA, which is considered to be constitutively expressed in human pancreatic adenocarcinoma, is rarely expressed in the biopsy specimens studied.

## List of abbreviations

CCK2R: Cholecystokinin-2 receptor; CCK2i4svR: Cholecystokinin-2 intron-4 retaining splice variant receptor; wt: wild-type (originally described); RT-PCR: reverse-transcription PCR; polyA^+^RNA: polyadenylated messenger RNA; aRNA: amplified RNA; ss-cDNA: single-stranded complementary DNA.

## Competing interests

The authors declare that they have no competing interests.

## Authors' contributions

AR, KB and HJM participated in the conception, study design and drafting of the manuscript. KB collected and selected the archival biopsy specimens in the study. AK and HJM were responsible for the acquisition, analysis and interpretation of data. All authors have read and approved the final manuscript.
